# Assessment of collaborative problem solving skills in Undergraduate Medical Students at Ziauddin College of Medicine, Karachi

**DOI:** 10.12669/pjms.341.13485

**Published:** 2018

**Authors:** Arsalan Manzoor Mughal, Sirajul Haque Shaikh

**Affiliations:** 1 Arsalan Manzoor Mughal, Assistant Professor, Department of Anatomy, Ziauddin University, Karachi, Pakistan; 2 Sirajul Haque Shaikh, Director, Department of Medical Education, College of Physicians & Surgeons, Karachi, Pakistan

**Keywords:** Problem-Based Learning, Problem Solving

## Abstract

**Objective:**

Collaborative Problem Solving Empirical Progressions from the Assessment and Teaching of 21st Century Skills (ATC21S) framework were used to determine the level of collaborative problem solving skills (CPS) in first, second and third year MBBS students at Ziauddin College of Medicine during Problem-Based Learning (PBL) sessions. Variations based on gender and roles were studied.

**Methods:**

It is an analytical comparative cross-sectional study in which seven PBL groups were selected per year by non-probability convenient sampling. Data was collected using the Collaborative Problem Solving Five Strands Empirical Progressions by the primary investigator through observation of the students during PBL sessions. Duration of study was six months.

**Results:**

We found that in our students, development of social dimension skills is facilitated to a greater extent than the development of cognitive dimension skills through the process of PBL. These skills are generally better developed in the leader compared to the scribe and members in a group. They are also more developed in females compared to males. Modification in them is also observed as the year's progress.

**Conclusion:**

Although PBLs facilitate development of CPS skills' progression however in our curriculum, PBLs mainly focus on social skills development and have less emphasis on cognitive skill development. Thus, hybrid instructional strategies with components from TBL and mentorship are recommended for better development of CPS skills.

## INTRODUCTION

Although traditional disciplines such as basic and clinical sciences have their place in medical education, there has been a shift towards the teaching of additional soft skills which will help the doctor of tomorrow work in a broader and more connected workplace.^1^ These are called 21^st^ century skills.^2^ Despite the recognition of their significance, only a few structured frameworks are available for assessment and development of these skills.^3^ The Assessment and Teaching of 21st Century Skills project (ATC21S) with contributions from 250 researchers from around the world is one of the most comprehensive among these frameworks. It provides comprehensive tasks for the assessment of 21^st^ century skills.^4^ One of these skills is Collaborative Problem Solving (CPS) which is defined as “working with others to solve a common challenge, including the contribution and exchange of ideas, knowledge or resources in order to achieve a shared goal”.^5^ CPS are ranked among the top five most essential skills required for success in college and the workforce.^6^ According to the ATC21S project, CPS can be classified into two dimensions - social skills and cognitive skills. The three elements of social skills include participation, perspective taking and social regulation whereas the two elements of cognitive skills are task regulation and knowledge building. These are called the five strands of CPS.^7^

Participation skills include the level of action, interaction and task completion. Perspective taking skills include responsiveness and audience awareness. Social regulation skills include metamemory, transactive memory, negotiation and initiative taking. Task regulation skills include problem analysis, goal setting, resource management and planning complexity. Knowledge building skills includes systematicity and information acquisition.^8^

The progression of each elemental CPS skill can be graded from A to F using the five-strand empirical projection frame work^7^ by observing the student in a collaborative problem-solving process. This assessment indicates the extent of development of each CPS skill element.^9^

Problem Based Learning (PBL)is an instructional method developed by Howard Barrows and Victor Neufeld at McMaster University, in which patient problems are used by the students to learn about basic and clinical sciences.^10^ Advantages of the PBL process include development of inter-disciplinary thinking, solution of complex tasks and reduction of cognitive overload by scaffolding provided by the facilitator.^11^ In addition, PBLs also facilitate learning of additional essential skills such as self-directed learning, epistemic practices and collaboration.^11^ Though these skills are not examined in tests, they are thought to be important for lifelong learning.^11^ However due to the lack of a proper assessment framework, it has also been claimed that it is unlikely that problem solving skills exist or if they can ever be learned by PBLs.^12^

The Ziauddin College of Medicine has an integrated modular hybrid curriculum with a mix of lectures, lab sessions as well as problem based sessions in the undergraduate medical program. It was the first college in the country to introduce PBLs back in 1996.^13^ The seven Jumps PBL process is carried out through MBBS years 1-3. Each MBBS batch is divided into 13 PBL Groups with 10 students in each to conduct PBLs. A PBL comprises of two Sessions each of two hours duration. Learning goals are generated at the end of Session one by completing Jumps 1-5 in a small group discussion format. These are researched by the students during self-study sessions as part of Jump 6. In Session two, students collaborate to discuss and achieve the set goals as part of Jump 7. Each session is facilitated by an instructor who is trained to conduct the process. Students are given roles during each session which include a leader, who directs the group, and a scribe, who takes notes. The remaining participants are called the members. These roles are rotational and change with each PBL.

Despite the claim that PBLs help develops CPS skills, no comprehensive framework was previously available to assess these skills. So, we used the ATC21S framework Collaborative Problem Solving Five Strands Empirical Progressions to determines the level of these skills in first to third year MBBS students and to find variations based on gender and roles.

## METHODS

In this comparative cross sectional study, seven PBL groups were selected per year by non-probability convenient sampling for adequate representation at a confidence interval of 95%, confidence level of 5%. After written informed consent, data was collected for each student individually regarding the social skills (participation, perspective taking, social regulation) in Session One and cognitive skills (task regulation, knowledge building) in Session Two using the five-strand empirical projection framework^7^ by observing the student in each collaborative problem-solving session. Each CPS skill was graded from A to F in the order of development as described in the framework. All data was collected by the primary investigator. Ethical approval was obtained from Ethics review committee of Ziauddin University prior to commencement of study.

Categorical data of CPS grades A-F was converted to numerical scores 1-6 respectively. Data was entered into SPSS and analysed by SPSS statistical software. 2-tailed independent samples student t test was used to compare mean scores of genders whereas analysis of variance (ANOVA) was used to compare mean scores of years 1-3 and roles of students. P-value less than 0.05 was considered as significant.

## RESULTS

Overall, the mean scores of the students were higher in social skill compared to cognitive skills. They scored highest in social regulation followed by perspective taking, participation, task regulation and knowledge building (Table-I).

**Table-I:** Mean and Standard Deviation of Collaborative Problem Solving Skills Scores.

Collaborative Problem Solving Skills		N	Mean	Standard Deviation
Social Skills	Participation	210	3.07	1.316
	Perspective Taking	210	3.09	1.347
	Social Regulation	210	3.10	1.364
Cognitive skills	Task Regulation	210	3.00	1.334
	Knowledge Building	210	2.92	1.326

**Fig. 1:**
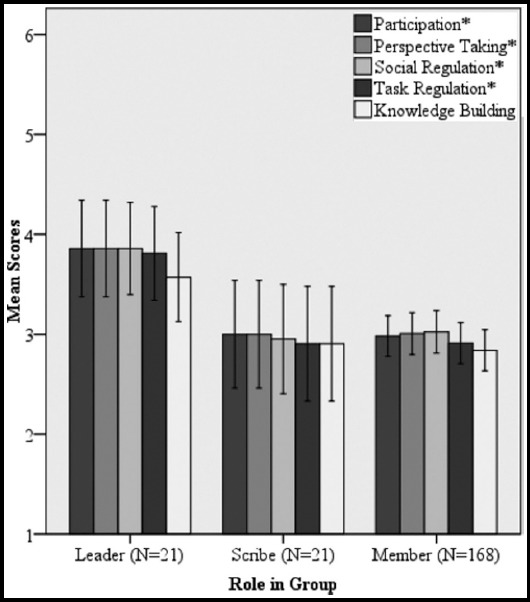
Comparison of mean collaborative problem solving skills scores in leader, scribe and member. Error bars indicate 95% confidence interval. * indicates p-value<0.05

There was a significant difference between the performance of leaders, scribes and members in all four skills except knowledge building. Mean scores were generally higher for the leaders compared to the scribes and members (Fig.1).

**Fig. 2:**
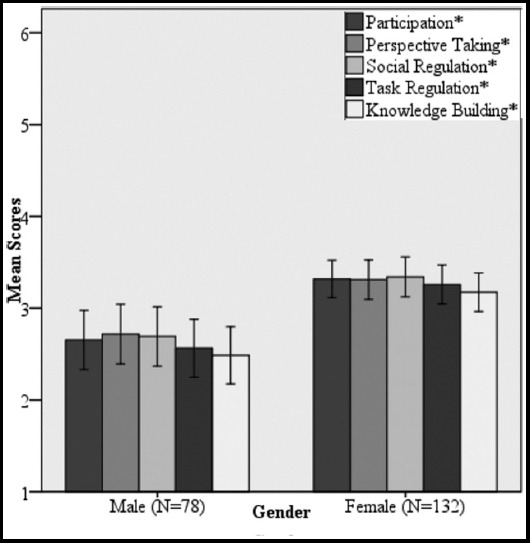
Comparison of mean collaborative problem solving skills scores in genders. Error bars indicate 95% confidence interval. * indicates p-value<0.05

A comparison of performance based on gender indicates that females got significantly higher scores in all skills compared to males. Among males, the strongest skill was perspective taking whereas females scored highest in social regulation (Fig.2).

**Fig. 3:**
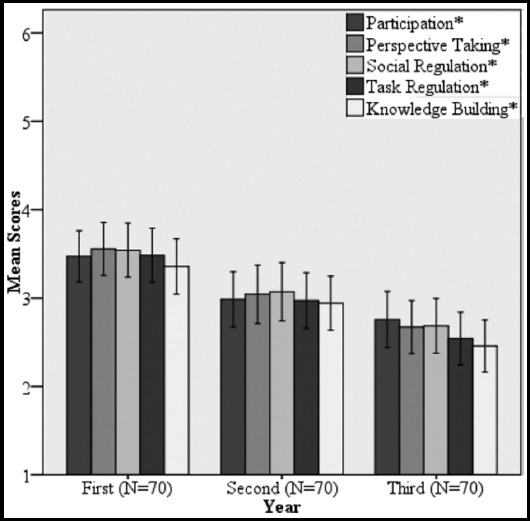
Comparison of mean collaborative problem solving skills scores in MBBS Year 1-3. Error bars indicate 95% confidence interval. * indicates p-value<0.05

A comparison between performances of different year batches indicates that the mean scores obtained in all five CPS skills were significantly higher in the first year MBBS batch and were progressively lower in the second and third year batches. The strongest skills in first year and third year students were perspective taking, social regulation and participation respectively. Knowledge building was the weakest skill in all three batches (Fig.3).

## DISCUSSION

Collaborative problem solving is an essential skill for the doctor of tomorrow.^14^ It is evident by the fact that collaboration and communication skills have been added to most competency frameworks of medical schools.^15^ Problem based learning is believed to develop these skills however to the best of our knowledge; this is the first study to assess these skills in our local context.

We found a mean score of around three in social and cognitive skills indicating “awareness of partnership” and “sharing & connecting information” in our student's respectively. The higher mean social skills scores in his study indicate that the process of PBL is encouraging their use. Ahonen et al.^16^ also reported that students ranked social skills and collaboration highest among the 21^st^ century skills. However, comparatively weaker scores in cognitive skills in our study highlights the relative weakness of the process in this dimension. Multiple studies have also reported that PBLs have no greater benefit in short term knowledge based test performance compared to traditional teaching methods.^17^,^18^ A recommendation could be use of additional components such as mandatory preassigned readings with individual and collaborative test solving. These are commonly used in Team Based Learning (TBL).^19^

Higher mean scores of CPS skills, especially in the social domain by the leaders compared to the scribes and members indicates greater use of CPS skills during the PBL. This is extremely positive as each student is given a chance on rotation to be a group leader, thereby encouraging them to use these skills. These skills will equip the students with the ability to manage and improve health care systems with a clear vision in a leadership role.^20^

We found better CPS skills scores in females compared to males, both in social and cognitive domains. Such gender differences have also been reported by other studies. Ahonen et al.^16^ reports that girls ranked social skills more highly whereas boys preferred technical skills. Development of social skills can be encouraged in males by reflection in mentorship programs.^21^

The significantly better performance of first year batch in all CPS skills domains and progressive decline in the subsequent years is worth noting. The reason for this could be a known problem that in a hybrid curriculum, there is inherent conflict with lectures which causes knowledge interference.^22^ In our college, lectures are taken by the senior faculty with greater subject knowledge and the PBLs are facilitated by the junior faculty with relatively less knowledge depth. Al Haqwi et al.^23^ suggests that the facilitator should possess both content and process expertise to get the best results from the PBL process. As the older students are self-regulated learners, they find lectures, demonstration and lab sessions more useful in this regard. Significance of traditional instructional strategies in this regard has also been reported by other studies from the region.^24^

The progressive change in the mean social skills score of batches indicates their modification with time. We noted a higher mean score of perspective taking in the first-year batch, indicating their effort to understand each other's points of view. The second-year batch has a higher mean score in social regulation, indicating their ability to resolve conflict. The third-year batch has a higher mean score in participation, indicating uniform contribution from the group. Social skills development is essential as process conflicts often turn into relationship conflicts when social skills are weak.^25^ Thus improved social skills enhance collaboration.

### Limitations of the Study

As this study was conducted in a single medical college, the results may reflect a local perspective and may be difficult to generalize. Also, being an observational study, the element of subjective bias cannot be ruled out. However, it was minimized by the fact that the data collection tool was highly structured and all data was collected by a single primary investigator who has an 8 years' experience of generating and conducting PBL sessions at the university and is a student of medical education. We couldn't generate a comparative group which is not exposed to PBL because all medical students undergo PBL teaching at the medical college in the basic health sciences. Only year 1-3 students could be included as the fourth and final year students don't have PBLs.

Knowing the levels of CPS skills in our students and their progression will help plan better PBLs and other teaching strategies to enhance their development. Teacher awareness and training will be essential in this regard.^26^

## CONCLUSION

PBLs facilitate development of CPS skills progression, as our students on average were aware of their partners and were able to share and connect information. It also facilitates development in leadership skills in the role of group leaders. However, in a hybrid curriculum, PBLs mainly focus on social skills development whereas traditional teaching methodologies retain their significance in cognitive skill development. Also, females have higher CPS skills compared to males in these PBL sessions. Hybrid instructional strategies with components from TBL and mentorship are recommended for better development of CPS skills.

### Authors' Contribution

**AMM:** Conception of research, acquisition, analysis and interpretation of data.

**SHS:** Critical revision for important intellectual content, final approval of the version to be published.
